# Associated Factors for Tuberculosis Recurrence in Taiwan: A Nationwide Nested Case-Control Study from 1998 to 2010

**DOI:** 10.1371/journal.pone.0124822

**Published:** 2015-05-01

**Authors:** Chung-Lin Hung, Jung-Yien Chien, Chih-Ying Ou

**Affiliations:** 1 Division of Hematological Oncology, Department of Internal Medicine, Dalin Tzu Chi Hospital, Buddhist Tzu Chi Medical Foundation, Chiayi, Taiwan; 2 Division of Chest Medicine, Department of Internal Medicine, National Taiwan University Hospital and National Taiwan University College of Medicine, Taipei, Taiwan; 3 Division of Chest Medicine, Department of Internal Medicine, National Cheng Kung University Hospital, College of Medicine, National Cheng Kung University, Tainan, Taiwan; Fundacion Huesped, ARGENTINA

## Abstract

**Background:**

The contribution of human immunodeficiency virus (HIV) co-infection to tuberculosis (TB) recurrence is well established worldwide. We conducted this study to investigate associated factors for recurrent TB in Taiwan, which has a relatively low prevalence of HIV.

**Methods:**

A case-control study nested within a nationwide population-based cohort was performed using the Taiwan National Health Insurance (NHI) database from 1998 to 2010. Patients with notified TB were identified according to diagnosis codes and prescriptions of anti-TB drugs for more than 60 days. Recurrent TB was defined as cases being retreated for more than 60 days and 6 months after the end of previous TB episode. Four controls were randomly selected from cohort and matched to each case by observational period within a calendar year. Socio-demographic variables and comorbidities were evaluated as factors associated with TB recurrence.

**Results:**

There were totally 760 patients being investigated (608 controls and 152 cases). During an average 5.12 years of follow-up, 3.76% of all developed recurrent TB and the incidence of TB recurrence was 734 per 100,000 person-years. About half of recurrence (55%) was notified within three years of follow-up, and most (86%) recurrences were intrapulmonary. Independent associated factors for TB recurrence included: male (odds ratio, OR: 2.23, 95% confidence interval, CI: 1.40–3.53), diabetes mellitus (DM) (OR: 1.51, 95% CI: 1.02–2.13), chronic obstructive pulmonary disease (COPD) (OR: 1.59, 95% CI: 1.08–2.36) and lower socio-economic status (p=0.001 between groups).

**Conclusions:**

Despite low prevalence of HIV in the Taiwanese population, the incidence of recurrent TB among Taiwanese was not less than that of other countries. Identification of subgroups such as male gender, low economic status, DM and COPD should be a high priority in TB control programs.

## Introduction

Recurrence of tuberculosis (TB) can be caused by reactivation from the same strain of *Mycobacterium tuberculosis* as in a previous TB episode, which is defined as relapse, or by reinfection with a different strain. Despite the availability of effective antimicrobial chemotherapy and implementation of Directly Observed Treatment, Short Course (DOTS), which was announced by the World Health Organization (WHO) in 1995 [1], recurrent TB remains a great concern to the global public health community [2]. In countries with a low or intermediate incidence of TB, the majority of recurrence is due to reactivation [3]. In contrast, in high-incidence countries, reinfection is the main reason for recurrence, mainly due to increased risk of exposure [3]. Apart from the variable incidences across areas with different endemic characteristics, a host of factors including patient immune and socio-economic status may also influence the likelihood of recurrent TB [4, 5].

The Centers for Disease Control (CDC) indicates Taiwan is an intermediate incidence setting for TB, with an incidence 54.5 cases per 100,000 population in 2012 [6]. Among host comorbidities, human immunodeficiency virus (HIV) co-infection is a well-known and independent risk factor for TB recurrence [7–10]. Nonetheless, only 0.8% of new TB cases in Taiwan occurred in HIV-positive patients, compared with 13% of TB cases in patients co-infected with HIV in the world [6]. Therefore, the relative contribution of recurrent TB to the overall annual TB incidence is likely to vary depending on the different epidemiological features of the area. Little research is reported on the incidence and causes of TB recurrence in Taiwan, and no national investigation has, so far, been conducted. A comprehensive understanding of TB recurrence would provide better efficacy of TB control and guidance for clinicians to identify patients at risk for TB recurrence. Thus, the aim of the present study was to investigate the incidence of recurrent TB and the associated factors that contribute to it in Taiwan.

## Materials and Methods

### Data Sources

National Health Insurance (NHI) is a mandatory universal health insurance program in Taiwan, providing comprehensive medical care to more than 95% of Taiwanese residents since 1996. The NHI sample files, managed by the National Health Research Institute (NHRI), comprise detailed utilization and demographic information for a randomly selected sample of one million NHI beneficiaries. These files include enrollment data, claims data, and a registry of drug prescriptions. This study used the dataset of one million nationwide representative population which contains patients’ identification numbers, gender, date of birth, dates of ambulatory visits, hospital admission and discharge data, medications, and diagnoses and procedures. Diagnoses and procedures were coded using the International Classification of Diseases, Ninth Revision, Clinical Modification (ICD-9-CM) format. All information was encrypted by the NHRI, which protected the anonymity of the patients while allowing specific patients to be selected for study and follow-up. The study met the patient confidentiality regulations of the Bureau of NHI. The study was approved by the institutional review board of National Chung Kang University Hospital (A-EX-103-005). According to the regulations of NHRI of Taiwan, only citizens of the Republic of China who fulfill the requirements of conducting research projects are eligible to apply for the National Health Insurance Research Database. As a result, our data availability is restricted by legal compliance. A contact email address (nhird@nhri.org.tw) to which requests for the data may be sent to.

### Study Design and Population

A retrospective, nested case-control study was conducted from January 1, 1998 to December 31, 2010, based on ambulatory care and inpatient discharge records. Our study cohort used the dataset of one million nationwide representative population insured in 2000, which is called as Longitudinal Health Insurance Database of 2000 (LHID 2000) and contains all medical records from 1996 to 2012. There were about twenty three million peoples in Taiwan, reported by Ministry of the Interior in 2014. Our study population comprised one twenty-third of the total, which could be representative statistically. All patients with a history of TB prior to 1998, HIV infection, and age less than 18 years were excluded. From January 1, 1998 to December 31, 2010, enrolled patients with the diagnosis of TB (ICD-9-CM codes 011–018) plus concurrent prescriptions for one (including fixed dose combination drugs of isoniazid / rifampin / pyrazinamide / ethambutol or isoniazid / rifampin) or at least two (including isoniazid, rifampin, pyrazinamide, or ethambutol) anti-TB drugs for more than 60 days were identified as newly diagnosed cases of TB. Being regarded as a mandatory communicable disease, Taiwan CDC set up a reporting system for TB control and surveillance. All suspects should be registered and finally notified once TB diagnosis was confirmed by physicians through bacterial/radiological/clinical evidence for TB. All notified TB patients should be treated with anti-TB drugs at least 6 months. By way of directly linking ICD-9-CM data and registry of drug prescription pooled in Taiwan NHI sample files, we could identify TB patients with definite notification confirmed by physicians. All enrollees were monitored until December 31, 2010, or until they developed recurrent TB, or were lost to follow-up (cancelled health insurance prior to December 31, 2010). Recurrent TB was defined as cases being retreated for more than 60 days and 6 months after the end of previous TB episode. Four control subjects with a history of TB but without recurrent TB were randomly selected from the enrollees and matched to each case of recurrent TB by observational period within a calendar year. In addition to investigating the factors associated with recurrent TB, we also compared TB locations between cases and controls by categorizing TB infection into intra- and extra-thoracic involvement according to the ICD-9-CM register (013–018 in the former one). The recurrent incidence with different follow-up periods after first TB diagnosis was calculated, too.

### Sociodemographic Variables and Comorbidities

Data for sociodemographic variables including age, gender, economic status, and residential area were obtained directly from the NHI files. We used the paid insurance premiums as a surrogate for household income level and socio-economic backgrounds, and further classified these into total five categories. Those with well-defined monthly wages were grouped into three categories: less than NT$ (New Taiwan dollar) 19200, NT$ 19200 to NT$ 30300, and NT$ 30300 or more. Those without well-defined monthly wages including local office beneficiaries, farmers and fishermen, veterans and low-income people who received total NHI’s nearly 100% coverage, were categorized as “fixed amount.” The latter fifth group, called “dependents”, was defined as subjects without available premium information. Residential area was divided into four locations: northern, central, southern, and eastern of Taiwan. Cases from the outlying islands were excluded. Patient comorbidities, based on the claims data, included diabetes mellitus (DM), dyslipidemia, hypertension, ischemic cardiovascular disease, cardiac arrhythmia, congestive heart failure, cerebrovascular disease, chronic obstructive pulmonary disease (COPD), asthma, bronchiectasis, silicosis, liver cirrhosis, end-stage renal disease, connective tissue diseases, and kidney transplant. Patients with HIV were excluded. Comorbidities were recorded when the selected ICD-9-CM codes were present in the claims data of enrollees for an average of more than three times per year during the observational period.

### Statistical Analysis

Data for continuous and categorical variables are presented as means ± standard deviations and number (%). Baseline differences between cases and controls were analyzed using the chi-square test or Fisher’s exact test for comparison of nominal variables. Student’s *t* test was used to analyze the differences between continuous variables. The trend in TB recurrence over time was analyzed using the Cochran-Armitage trend test. Associated factors for TB recurrence were analyzed using a logistic regression. Multivariate analysis was verified after adjusting for potentially confounding factors in univariate analysis and the result was represented as odds ratio (95% confidence intervals). A p-value of <0.05 was considered significant. Data extraction and statistical analyses were performed using SAS 9.3 (SAS Institute Inc., Cary, NC, USA).

## Results

### Baseline Characteristics of Study Cohort

**Fig 1:**
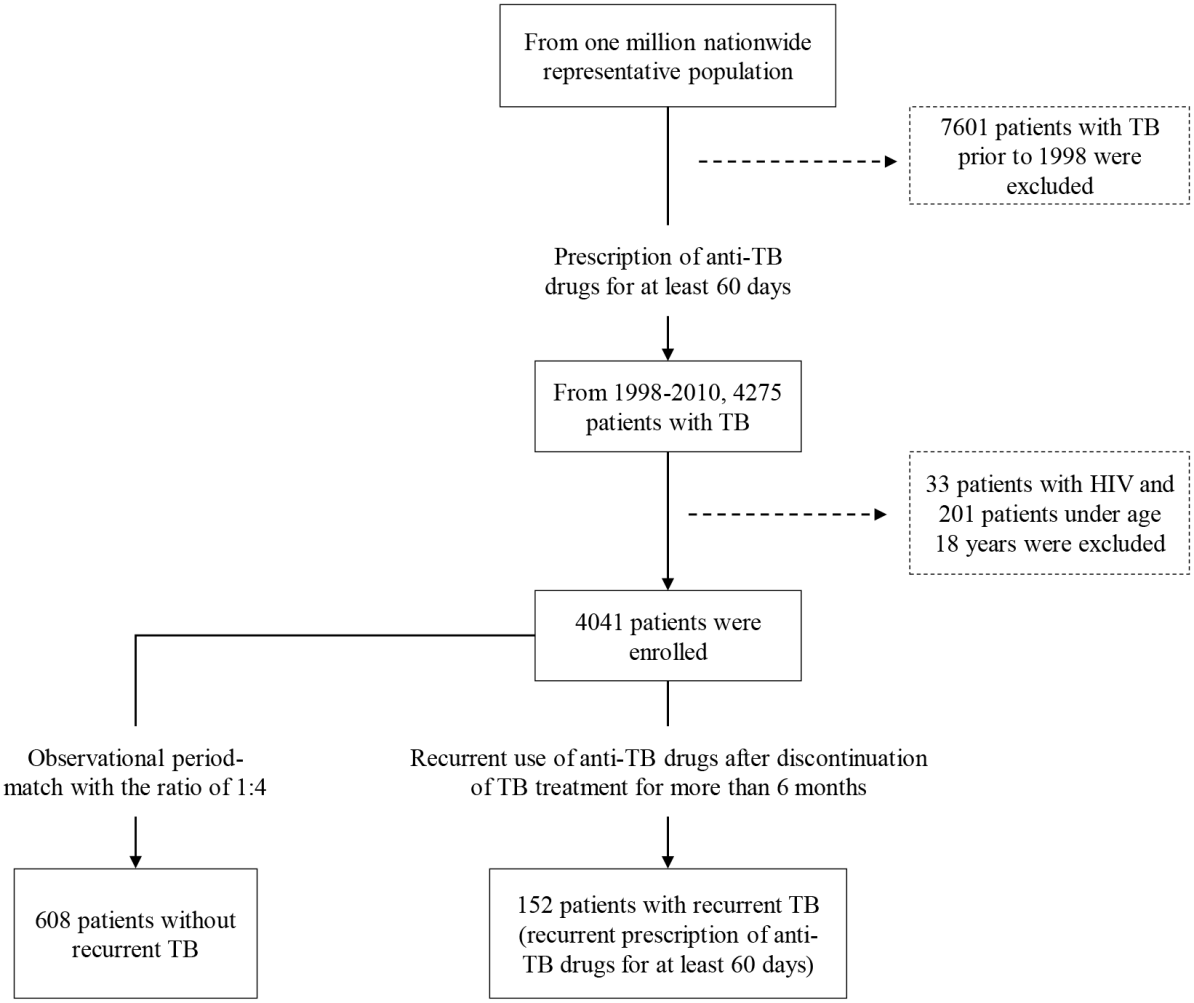
Flow diagram for the enrollment of study participants in the nested case-control study. After excluding patients with antecedent TB prior to 1998, HIV infection and those less than 18 years of age, the numbers of nested cohort, case and control were 4041, 152 and 608, respectively.

The flowchart for patient selection is illustrated in Fig 1. From January 1, 1998 to December 31, 2010, after excluding patients with antecedent TB prior to 1998, HIV infection and those less than 18 years of age, there were 4041 patients with new diagnoses of TB who received anti-TB treatment and the average observational period was 5.12 ± 3.44 years. Among these enrollees with a history of TB, 152 patients (3.76%) had recurrent TB during the observational period and the incidence of TB recurrence was 734 per 100,000 person-years. 608 observational period-matched control patients from the remaining cohort were matched to each case of recurrent TB (n = 152) (Fig 1). Table 1 shows aging, male, lower socio-economic status, DM and COPD were the significantly higher in the recurrent TB group. Eastern Taiwan had the highest TB incidence and recurrence rates, which was similar to a previous study result [11].

**Table 1:** Characteristics of control and case groups.

	Recurrence^-^	Recurrence^+^	p
	(n = 608)	(n = 152)	
Age	51.95 ± 18.71	56.94 ± 16.40	**0.001**
Gender			
Female (%)	216 (36)	28 (18)	-
Male (%)	392 (64)	124 (82)	**< 0.0001**
Socio-economic status			
NT$ 30300 or more	94 (16)	11 (7)	**-**
NT$ 19200 to NT$ 30300	245 (40)	66 (43)	**0.01**
Less than NT$ 19200	33 (5)	10 (7)	**0.04**
Dependents	131 (22)	30 (20)	0.07
Fixed amount	105 (17)	35 (23)	**0.004**
Residential area			
Northern	109 (18)	26 (17)	-
Middle	128 (21)	31 (20)	0.96
Southern	152 (25)	33 (22)	0.75
Eastern	219 (36)	62 (41)	0.51
Comorbidities, n (%)			
Diabetes	121 (20)	44 (29)	**0.02**
Dyslipidemia*	77 (13)	14 (9)	0.24
Hypertension	171 (28)	48 (32)	0.40
Ischemic cardiovascular disease	89 (15)	17 (11)	0.27
Cardiac arrhythmia*	35 (6)	5 (3)	0.22
Congestive heart failure	30 (5)	10 (7)	0.42
Cerebrovascular disease	67 (11)	20 (13)	0.46
Chronic obstructive pulmonary disease	227 (37)	81 (53)	**0.0003**
Asthma	80 (13)	24 (16)	0.40
Bronchiectasis	59 (10)	19 (13)	0.31
Silicosis*	10 (2)	5 (3)	0.20*
Immuno-compromised diseases^&^	5 (1)	5 (3)	0.20*

^**-**^ **Without recurrence**

^**+**^ **With recurrence**

*Fisher’s exact test for comparison of nominal variables.

^&^Immuno-compromised diseases included liver cirrhosis, end-stage renal disease, kidney transplant and connective tissue disease.

### Locations of TB Involvement in Patients With and Without Recurrence

Table 2 compares the sites of intra- and extra-thoracic TB involvement among the patients with and without recurrence. Whether there was a recurrence or not, intra-thoracic TB accounted for > 90% of all cases. Pulmonary TB was the predominant type both in the control and recurrent group (Table 2).

**Table 2:** Site of tuberculosis (TB) involvement among patients with and without TB recurrence.

Locations of TB	Recurrence^-^	Recurrence^+^	p
	(n = 608)	(n = 152)	0.44
Intra-thoracic, n (%)	640 (94)	161 (95)	0.15
Primary	23 (3)	2 (1)	
Pulmonary	579 (85)	145 (86)	
Pleurisy	38 (6)	14 (8)	
Extra-thoracic, n (%)	43 (6)	8 (5)	0.67
Central nervoussystem	2 (< 1)	1 (1)	
Peritoneal	6 (1)	1 (1)	
Bone/Joint	10 (1)	0 (0)	
Genitourinary	6 (1)	1 (1)	
Cutaneous	14 (2)	4 (2)	
Miliary	5 (1)	1 (1)	

^**-**^ **Without recurrence**

^**+**^ **With recurrence**

### Duration and Associated Factors for TB

**Fig 2:**
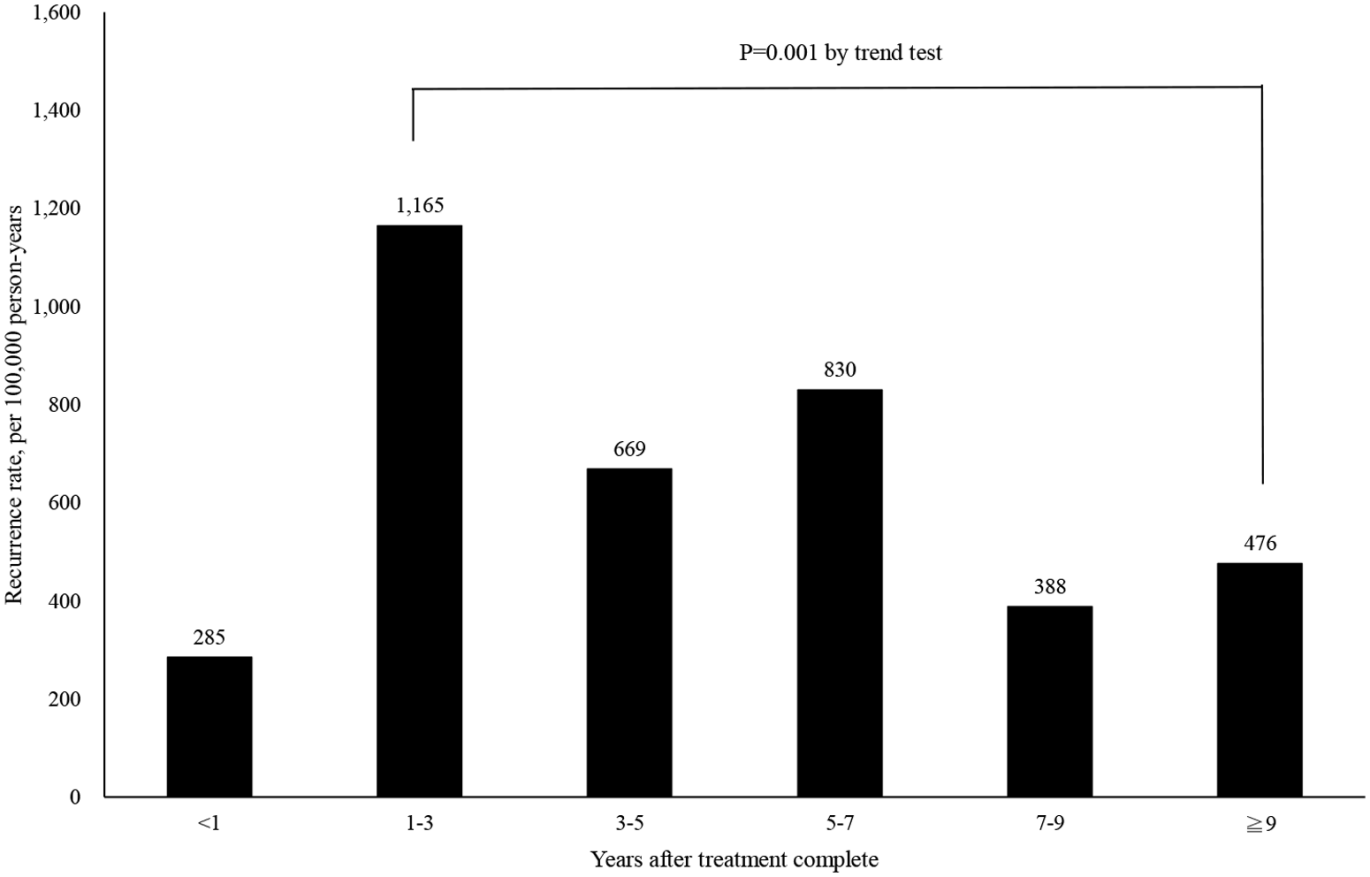
Incidence of recurrent tuberculosis among 4041 patients being treated for *Mycobacterium tuberculosis* infections. The incidence peaked within 1 to 3 years after treatment complement and gradually declined from trend analysis.

The average duration of recurrence was 3.54 ± 2.53 years (minimum: 0.63 years, maximum: 12.76 years). More than half of the recurrent TB patients (55%) were diagnosed within three years after the first TB episode (Table 3). As illustrated in Fig 2, the incidence of TB recurrence was highest within one to three years post TB treatment (incidence: 1165/100,000 person-years) and gradually decreased over time according to the trend analysis (p = 0.001). Age, gender, socio-economic status, and comorbid diseases were included in our analysis using univariate and multivariate logistic regression. Finally, the following variables were independent associated factors for TB recurrence: male gender (OR: 2.23, 95% CI: 1.40–3.53), DM (OR: 1.51, 95% CI: 1.02–2.13) and COPD (OR: 1.59, 95% CI: 1.08–2.36) (Table 4). Compared with the group with the highest household income level (NT$ 30300 or more), patients with lower socio-economic status were independently at risk for recurrent TB (fixed amount: OR: 3.40, 95% CI: 2.35–5.22, less than NT$ 19200: OR: 2.99, 95% CI: 1.82–3.97, NT$ 19200 to NT$ 30300: OR: 2.15, 95% CI: 1.06–4.36) (Table 4).

**Table 3:** Time to tuberculosis recurrence by year since follow-up.

Period of follow-up	Person-years of follow-up	No. of events	Total recurrence (%)	Incidence
< 1 year	3860	11	7	285
1–3 years	6268	73	48	1165
3–5 years	4486	30	20	669
5–7 years	3012	25	16	830
7–9 years	1804	7	5	388
> 9 years	1261	6	4	476

**Table 4:** Logistic regression analysis of predictors for tuberculosis recurrence.

	Univariate		Multivariate	
	Odds ratio	p	Odds ratio	p
	(95% CI)		(95% CI)	
Age (years)				
18–29		-		
30–59	1.89 (0.98–3.67)	0.06	1.67 (0.82–3.39)	0.16
> 60	**2.50 (1.30–4.79)**	**0.006**	1.68 (0.81–3.47)	0.16
Gender				
Female		-		
Male	**2.44 (1.57–3.80)**	**< 0.0001**	**2.23 (1.40–3.53)**	**0.001**
Socio-economic status				
NT$ 30300 or more		-		
Fixed amount	**3.93 (2.03–6.58)**	**0.01**	**3.40 (2.35–5.22)**	**0.03**
Dependents	1.96 (0.93–4.10)	0.08	2.09 (0.94–4.62)	0.07
Less than NT$ 19200	**2.59 (1.01–6.65)**	**0.05**	**2.99 (1.82–3.97)**	**0.03**
NT$ 19200 to NT$ 30300	**2.30 (1.17–4.55)**	**0.02**	**2.15 (1.06–4.36)**	**0.04**
Comorbidities				
Diabetes	**1.86 (1.27–2.25)**	**0.02**	**1.51 (1.02–2.13)**	**0.03**
Dyslipidemia	0.70 (0.38–1.27)	0.24		
Hypertension	1.18 (0.80–1.73)	0.40		
Ischemic cardiovascular disease	0.73 (0.42–1.28)	0.27		
Cardiac arrhythmia	0.56 (0.21–1.45)	0.23		
Congestive heart failure	1.25 (0.82–1.91)	0.42		
Cerebrovascular disease	1.22 (0.72–2.09)	0.46		
Chronic obstructive pulmonarydisease	**1.92 (1.34–2.74)**	**< 0.0001**	**1.59 (1.08–2.36)**	**0.02**
Asthma	1.24 (0.75–2.03)	0.40		
Bronchiectasis	1.33 (0.77–2.31)	0.31		
Silicosis	2.03 (0.69–6.04)	0.20		
Immuno-compromised diseases^&^	1.71 (0.77–8.31)	0.20		

^&^Immuno-compromised diseases included liver cirrhosis, end-stage renal disease, kidney transplant, and connective tissue disease

## Discussion

The study findings estimated the overall incidence of recurrent TB in Taiwan to be 734 cases per 100,000 person-years from 1998 through 2010. Although there is a low prevalence of HIV infection in Taiwan estimated at 0.2% in 2012 [12], patients with recurrent TB still pose a potential public-health threat in a setting with an intermediate incidence of TB (54.5 cases per 100,000 population in 2012) [6]. We found that the gender (male predominance) and host comorbidities (DM and COPD) were significantly associated with TB recurrence. TB recurrence peaked one to three years after the initial diagnosis at 1165 cases per 100,000 person-years, and the majority of cases (55%) occurred within three years of follow-up. Lower socio-economic status was also an independent factor in predicting recurrent TB.

A systemic review of observational studies and controlled trials calculated a median (range) recurrence rate of 10,310 (0–18,940) episodes per 100,000 person-years in high incidence countries (defined as national incidence of at least 100 per 100,000 population) and 2380 (1000–5900) episodes per 100,000 person-years in low incidence settings, at 6 months following completion of treatment regimens [13]. However, among HIV-specific studies, the overall TB recurrence rate was higher among HIV-infected individuals (6.7% in HIV-infected and 3.3% in non-HIV infected) [13]. This was closer to our recurrence rate (3.76%) in our defined TB cohort without HIV infection. Except for HIV infection, other risk factors included poor treatment adherence [14], South Asians [8], male gender, immigrant status, intravenous drug use, smoking tobacco, drinking alcohol, and being in prison [7]. Consistent with these study results, we also found that male gender and lower economic status were predictors of TB recurrence, implying that smoking and poor nutrition could be potentially triggers. As a result of long observational period covering across the initiation of DOTS in Taiwan (since 2006), we could not evaluate the impact of DOTS on the treatment success in our study. From a further analysis, we grouped our recurrent cases according to time of recurrence by calendar year during study period, the annual distribution of recurrent number was 1, 3, 9, 9, 11, 14, 15, 15, 16, 15, 15, 14, and 15 from 1998 to 2010. This trend was gradually stationary and not affected by DOTS intervention. Furthermore, we also calculated the annual TB relapse rate from Taiwan CDC website (3.70% in 2010 TB cohort), which was similar to our recurrent rate (3.76%) during the study period. We speculated that the effect of DOTS may not be the essential factor, but in part, in the TB recurrence of our study. As we know, the emergence of multidrug resistant TB (MDR-TB) is substantially decreased after DOTS implementation [15], without inclusion of MDR-TB patients in our study design might be the reasonable cause.

Our study is the first nationwide-based assessment of recurrent TB in Taiwan and covered a long longitudinal period from 1998 to 2010. Given the low-to-moderate incidence of TB in Taiwan, we concluded the incidence of recurrent TB in Taiwan did not differ from the result demonstrated by Millet et al during 2003–2009 in Spain, who concluded 13 times higher than the incidence of TB in the general population (341 recurrent cases per 100,000 person-years versus 26.3 notified cases per 100,000 person-years) [10]. Nonetheless, according to co-epidemics of TB and HIV reported by the WHO in 2012, the proportion of TB patients co-infected with HIV in Spain (8.9%) was higher than in Taiwan (0.8%) [6]. This indicates different contributors are leading to TB recurrence in Taiwan. We found that DM and COPD were independently associated with recurrent TB. A nationwide cohort study also using NHI data from Taiwan reported that higher doses of oral corticosteroids and oral β-agonists were significant contributors to pulmonary TB in COPD patients (hazard ratio, HR: 1.17 vs. 1.05, 95% CI: 1.03–1.32 vs. 1.01–1.09, respectively) [16]. These two classes of respiratory drugs were not first-choice therapies for the management of COPD, and thus, were regarded as add-on therapies in acute, exacerbated, or severe disease groups. We reasoned that a more extensive TB burden caused by attenuating adaptive immunity in these frequently exacerbated COPD patients could have resulted in higher risk for recurrent TB. Another recent study [17] investigated the association between DM and TB relapse among Taiwanese during 2006–2007 and revealed a significant association (HR: 1.96, 95% CI: 1.22–3.15). However, the observed large effect in that study could be overestimated, having been caused by uncontrolled confounding variables such as COPD, which was also a risk factor for pulmonary TB [16].

Recurrence of TB is associated with increased risk of drug resistance and higher mortality. Before DOTS became the norm, epidemiological studies investigating the factors associated with recurrent TB showed that compliance was a strong predictor of recurrent disease [18]. Even so, a recent meta-analysis showed that directly observed therapy was not superior to self-administered therapy in preventing microbiologic failure, relapse, or acquired drug resistance [19]. In our study, the majority of recurrent cases were identified within three years, implying that endogenous reactivation was more likely to occur in the initial period after treatment completion [20]. We speculated that impaired host immunity in patients with DM or COPD was associated with greater severity of the first TB episode (as shown radiologically or bacteriologically) and ultimately higher recurrent rate for TB [21]. Whether inadequate treatment such as an inappropriate regimen or treatment duration is the main cause for relapse in patients with DM or COPD requires further investigation.

There are some limitations to our study. First, observations were retrospectively based on diagnostic codes and prescription history. Therefore, we were unable to identify bacteriological or radiological differences that could have potentially affected the severity of the first TB episode among the patients with recurrent TB. Furthermore, the absence in genotyping of recurrent isolates may hinder us to differentiate either a true relapse or a new episode of TB caused by reinfection. Second, because there were few patients in the recurrent TB group, further information associated with the control status of comorbidities were inconclusive and limited based on the NHI claims data. Third, the lack of a consistent definition of recurrence across all studies may complicate interpretation of our findings. External validity of our conclusion through a prospective cohort study design could be more powerful in guiding clinical practice. Finally, other variables such as drugs abusers or alcohol could not be identified from our database and thus we could not conclude the association with TB recurrence.

In conclusion, we conducted a national investigation of recurrent TB in Taiwan, taking a long-term observational view from 1998 to 2010. The results reinforce the notion that male gender, low economic status, and comorbidities such as DM and COPD are high-risk populations for recurrent TB. Further studies are required to evaluate the effectiveness of extended treatment courses or individualized post-treatment follow-up strategies for the prevention of TB recurrence and transmission.
